# Supplementary figures and images for: Fold or hold: experimental evolution in vitro
Source: J Evol Biol. 2013 Sep 4;26(10):2123–34. doi: 10.1111/jeb.12233 (PMC4274015; doi:10.1111/jeb.12233)

**
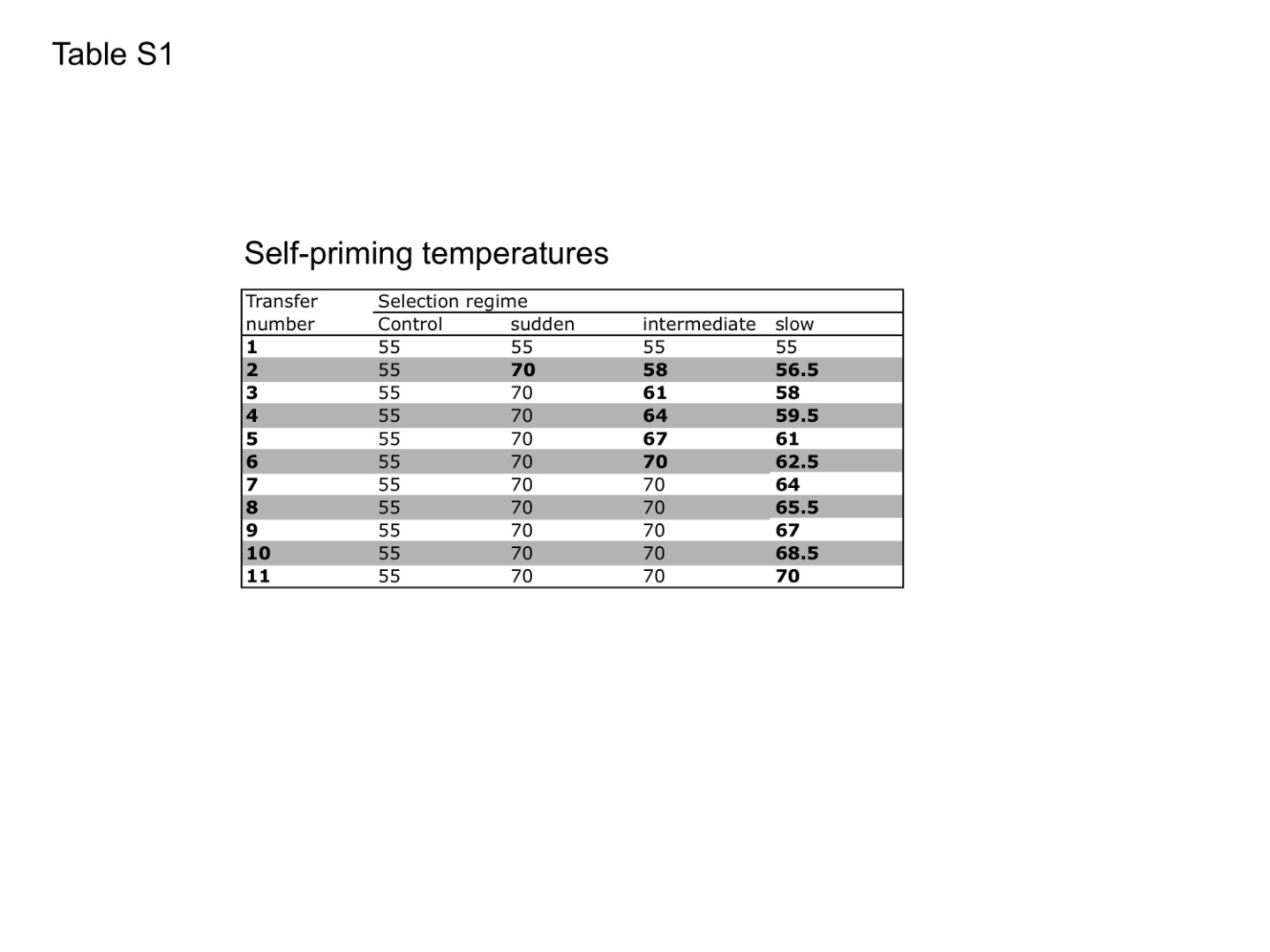
**

Supplement: Supplementary file 1 — Table S1 Temperatures in °C used for the self-priming step at each transfer for each selection regime. [file jeb0026-2123-sd1.docx]

**
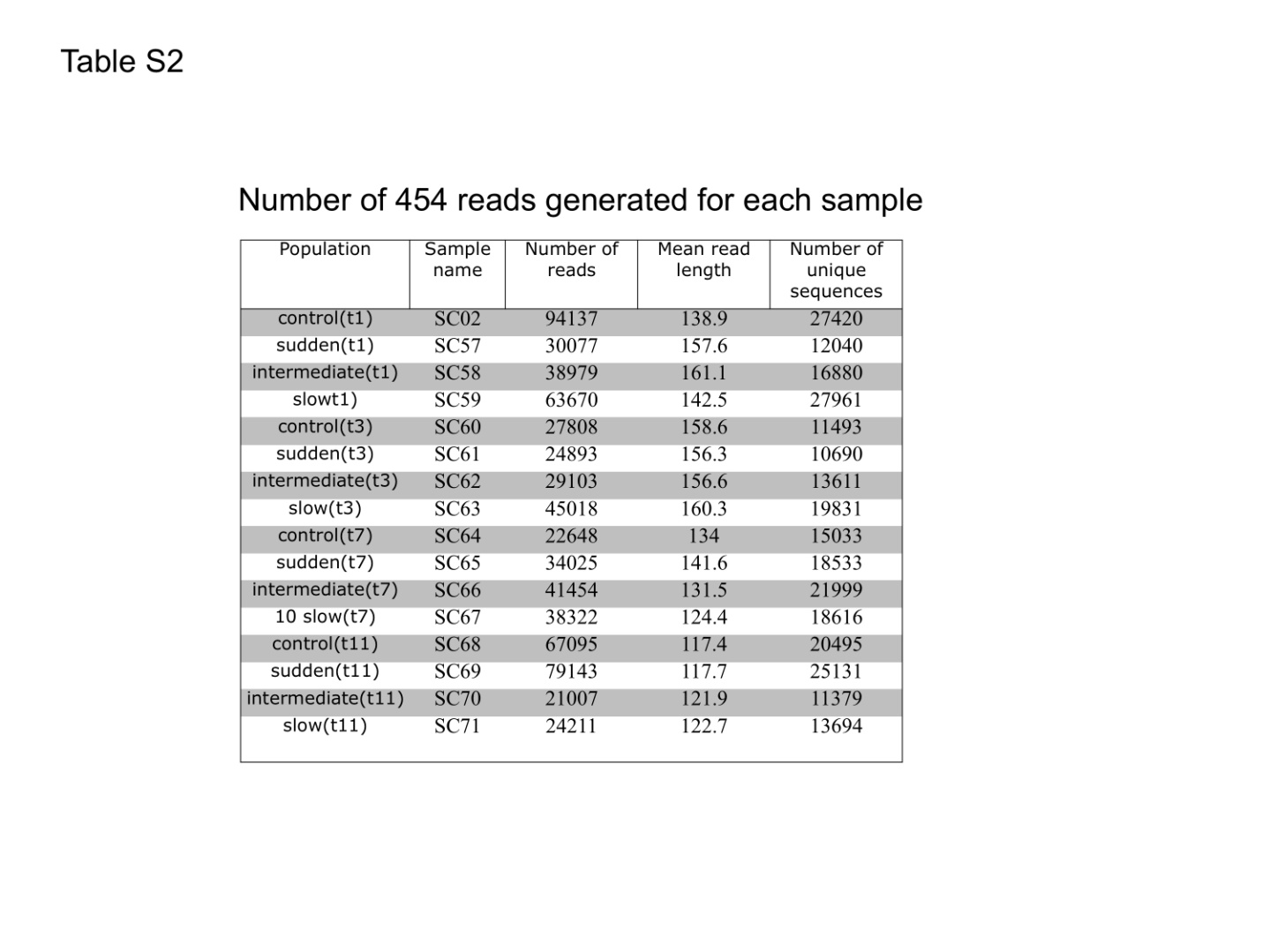
**

Supplement: Supplementary file 2 — Table S2 Number of 454 reads generated for each sample. [file jeb0026-2123-sd2.docx]
